# Understanding the Complexities and Changes of the Astronaut Microbiome for Successful Long-Duration Space Missions

**DOI:** 10.3390/life12040495

**Published:** 2022-03-28

**Authors:** Donatella Tesei, Anna Jewczynko, Anne M. Lynch, Camilla Urbaniak

**Affiliations:** 1 Department of Biotechnology, University of Natural Resources and Life Sciences, 1190 Vienna, Austria; donatella.tesei@boku.ac.at; 2 Department of Biology, University of Waterloo, Waterloo, ON N2L 3G1, Canada; aejewczy@uwaterloo.ca; 3 Department of Pulmonary Medicine, The University of Texas MD Anderson Cancer Center, Houston, TX 77030, USA; anne.lynch@bcm.edu; 4 Graduate Program in Developmental Biology, Baylor College of Medicine, Houston, TX 77030, USA; 5 ZIN Technologies Inc., Middleburg Heights, OH 44130, USA; 6 NASA Jet Propulsion Laboratory, California Institute of Technology, Pasadena, CA 91109, USA

**Keywords:** microbiome, spaceflight, space biology, astronaut, human exploration

## Abstract

During space missions, astronauts are faced with a variety of challenges that are unique to spaceflight and that have been known to cause physiological changes in humans over a period of time. Several of these changes occur at the microbiome level, a complex ensemble of microbial communities residing in various anatomic sites of the human body, with a pivotal role in regulating the health and behavior of the host. The microbiome is essential for day-to-day physiological activities, and alterations in microbiome composition and function have been linked to various human diseases. For these reasons, understanding the impact of spaceflight and space conditions on the microbiome of astronauts is important to assess significant health risks that can emerge during long-term missions and to develop countermeasures. Here, we review various conditions that are caused by long-term space exploration and discuss the role of the microbiome in promoting or ameliorating these conditions, as well as space-related factors that impact microbiome composition. The topics explored pertain to microgravity, radiation, immunity, bone health, cognitive function, gender differences and pharmacomicrobiomics. Connections are made between the trifecta of spaceflight, the host and the microbiome, and the significance of these interactions for successful long-term space missions.

## 1. Introduction

Humans have been exploring space for the last sixty-five years and, with the creation of the International Space Station, have been living and working in space continuously for the past 21 years. Astronauts endure many physiological and psychological changes while in space because of altered gravity, radiation, and confinement, to name but a few factors. While some spaceflight side effects are well known, such as bone loss [1], muscle atrophy [2], altered ocular structure [3], cognitive decline [4], fluid redistribution [5] and immune dysregulation [6], others, such as the taxonomic and functional changes of the astronaut microbiome, have been less studied, with the impact on astronaut health even less so. Understanding how the human microbiome adapts to space travel and how this influences astronaut health, pre-, post- and in-flight, is essential in reaching our goal of long-duration human exploration in low Earth orbit (LEO) and beyond. This review will discuss changes in the astronaut microbiome as a result of spaceflight (and other stressors pre- and post-flight), how these changes may impact astronaut health, and the resulting consequences for long-duration spaceflight. We also provide recommendations for industry and government entities designing future manned missions to the Moon and Mars on how to incorporate microbiome data into their planning and ways in which the microbiome can be targeted or manipulated to ensure successful long-duration human exploration beyond low Earth orbit.

## 2. Human Microbiome

Our body consists of trillions of bacteria that are on par with the number of human cells we have [7]. The gastrointestinal tract (GIT) alone harbors 100 trillion bacteria, consisting of 1000 different species, 7000 strains and 3.3 million non-redundant microbial genes [8,9,10]. The skin bacterial communities on a typical hand consist of >150 species, with only 13% similarity amongst different individuals [11]. Other sites have simpler bacterial communities, like the healthy vagina, which is mainly composed of *Lactobacillus* [12]. Human breast tissue, once thought of as sterile, is now accepted as having a stable microbial population [13,14]. These microbial communities, found in and outside our body, are referred to as the “human microbiome”, a term first coined in 2001 by Joshua Lederberg to refer to the “ecological community of commensal, symbiotic and pathogenic microorganisms that literally share our body space” [15]. These microorganisms consist of archaea, eukaryotes (fungi and protists) and viruses, with bacteria making up the majority of organisms present (99%) [10]. While the terms “human microbiome” and “human microbiota” are often used interchangeably, the latter refers to the microbial taxa associated with humans, while the former refers to the collection of microbial taxa and their genes [16].

The microbial communities that colonize various parts of our body are important in promoting health, by synthesizing vitamins the host cannot make, salvaging energy from indigestible compounds, creating a competitive environment to prevent pathogen colonization, promoting maturation and regulation of the immune system, contributing to vascular development and angiogenesis or enhancing the integrity of the epithelial barrier [17,18,19,20,21,22,23]. These benefits are achieved by a delicate balance of commensals, symbionts and pathobionts that collectively make up one’s microbiome. If this balance is disrupted even slightly, a breakdown in homeostasis will occur, leading to disease [24]. Microbial differences have been documented between healthy and diseased individuals with periodontitis [25], inflammatory bowel disease [26], psoriasis [27], asthma [28], bacterial vaginosis [29], colorectal cancer [30] and breast cancer [31]. These observed microbial differences are not simply a consequence of the diseased state creating an environment that selects for certain bacteria, as studies have shown that healthy animals transplanted with feces from those with obesity [32], colitis [33] and colorectal cancer [34] then go on to develop disease. Shifts in bacterial profiles not only have consequences at the site of origin but can have distal site effects as well. For example, alterations in the gut microbiota can have effects on the brain [35,36], liver [37,38], and pancreas [39], while microbial shifts in the oral cavity can be detrimental to cardiovascular health [40].

## 3. Spaceflight and the Astronaut Microbiome

### 3.1. Spaceflight Hazards: Conditions and Challenges Facing Space Travelers

**Figure 1:**
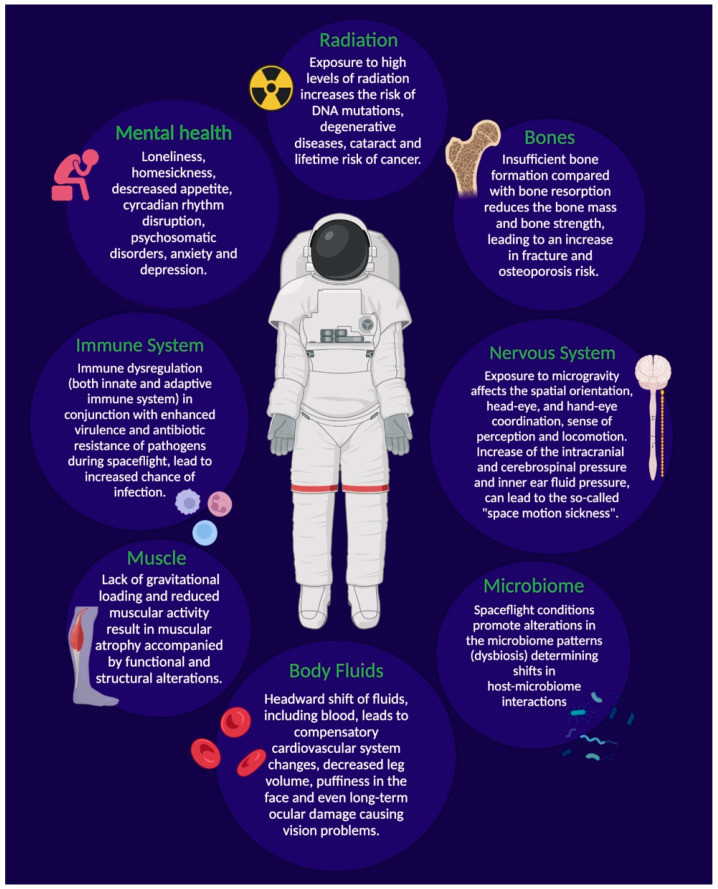
**Biological features of spaceflight.** In space, microgravity, radiation, and confinement in a closed environment thousands of miles away from Earth pose health risks and drive many physiological changes and psychological effects seen during spaceflight. Figure created with BioRender.com (accessed on 18 February 2022).

Long-term spaceflight and especially deep-space exploratory missions represent an extreme environment for humans that demands adaptations to both physical and psychological stressors (Figure 1) [41]. As such, a large part of spaceflight research focuses on the effects of space exposure on humans, with the aim to elucidate physiological, psychological and behavioral health risks and the necessary solutions to combat them [42]. Much of the existing knowledge about spaceflight hazards derives from crewed missions in LEO, such as those onboard the International Space Station (ISS), and analog missions on Earth. Additionally, a significant amount of data have been generated by experiments carried out in ground-based facilities, simulating different space conditions. This information, however, may provide inaccurate estimates into the risks connected to deep-space missions (outside LEO), which involve more time spent in space during a single mission than that hitherto experienced by astronauts (i.e., on average 7 months). Moreover, deep-space exploration will venture beyond the protective effects of Earth’s geomagnetic field; therefore, crewmembers on upcoming missions to the Moon, Mars and beyond will be exposed to significantly higher doses of cosmic radiation. 

#### 3.1.1. Radiation

The global radiation dose for astronauts is affected by galactic cosmic rays (GCRs) originating from outside the solar system, which include high atomic number and energy (HZE) ions and high-intensity solar particle events (SPEs) that produce pulses of heavy ion and energetic proton radiation [43]. It is estimated that a one-year stay on the lunar surface would result in absorbed doses for crews in the range from 100 to 120 mGy (milligray), whereas during a three-year Mars mission, transit and stay included, the values would increase to 450 mGy [44]. In comparison, the predicted doses for 6- to 12-month ISS missions range instead from roughly 30 to 120 mGy [44]. For reference, 4.5 Gy has been indicated as the LD_50_ value for human cells (median lethal dose) [45]. During SPEs, astronauts may additionally be exposed to an extremely high dose of protons (i.e., up to 1 Gy or more) that could lead to acute radiation sickness (ARS) [46,47]. Although radiation derived from SPEs is effectively absorbed by the shielding material of a spacecraft or well-designed spacesuit, not all SPEs are predictable and can therefore pose a real threat to astronauts, especially during planetary extravehicular activity (EVA), which would take place more frequently than during a 6-month ISS stay [48]. 

The consequences of chronic exposure to radiation, and to CGR in particular, is concerning, due to the high energy, high penetrability and ionizing nature of their most hazardous components, the HZE particles [49]. These particles are so penetrating that shielding can only partially protect against exposure and the large emission of secondary neutrons that may follow, posing an additional hazard to the crew [50]. The types of radiation encountered in space are different from terrestrial radiation, such as X-rays and gamma rays, and induce distinct patterns of DNA double-strand breaks and disease outcomes, resulting in epigenetic changes and persistently high levels of oxidative damage and tissue inflammation following exposure [51]. This is relevant because of the association of oxidative stress with the etiology of several human diseases, including cancer, cardiovascular and neurodegenerative disorders (e.g., altered sensory perception, neurovestibular problems, etc. [52]), gastrointestinal diseases [53], and the possible correlation between spaceflight and the onset of a number of diseases normally related to aging [54]. Exposure to space radiation, especially when considering that heavy ions are more effective per unit dose in causing solid cancer compared to gamma-rays [55], can therefore have a detrimental impact on the quality of life during and post flight. Visual disturbances appear to also be correlated to radiation exposure, as indicated by reports from past Apollo, Skylab and MIR missions where astronauts experienced flashes of light moving across their visual field, possibly due to alteration in perception caused by ionizing radiation [56]. An additional aspect to consider when assessing radiation-related risks are the non-targeted effects (NTEs) of radiation, which increase biological effectiveness for low doses of high linear energy transfer radiation (LET), such as HZE particles, and may lead to alterations in cell signaling and/or genomic instability of cell progeny [55,57]. 

#### 3.1.2. Microgravity

Along with radiation, microgravity—a condition in which the gravity level is almost zero but not neutralized—represents another major health hazard related to spaceflight. While astronauts mostly experience microgravity during spaceflight and onboard the ISS, they do experience intermediate periods of hypergravity (e.g., 3–6 G) during launch and ascent and upon descent back through the Earth’s atmosphere [58]. Additionally, various levels of altered gravity are found on other planetary bodies, such as on the Earth’s Moon—one-sixth gravity (G)—and Mars—one-third gravity (G)—(hypogravity) [42]. 

Spaceflight and ground-based analog experiments have shown that altered gravity, as well as the transition through various levels of gravity, subject biological systems (i.e., humans, animals, plants) to varying levels of stress with negative consequences. Specifically, microgravity can induce cellular and molecular alterations with changes to the genome, epigenome, and proteome, connected with a range of pathologies [42]. In humans, exposure to microgravity can influence several body systems such as the neurovestibular, cardiovascular, musculoskeletal, bone metabolic and immune-hematological system [59,60]. For example, altered body fluid distribution occurs during microgravity and this headward shift of fluids, including blood, leads to a compensatory cardiovascular system change, with increased intracranial pressure, cerebrospinal pressure or inner ear fluid pressure, decreased leg volume, puffiness in the face and even long-term ocular damage [61]. According to the “fluid shift” theory, this increased pressure is the root of “space motion sickness”, a set of symptoms that impair operational performance of 60–80% of astronauts. Additional interconnected abnormalities include morphological changes in the white and grey matter of the brain following long-duration spaceflight (average of 171 days), which have been attributed to the structural neuroplasticity of the sensorimotor system, in an effort to adapt motor strategies to maintain physiological homeostasis and ensure proper behavioral output in space [3]. Lack of gravitational loading also results in muscle atrophy, especially in the lower extremities, accompanied by functional and structural alterations. While muscular loss has been linked to reduced muscular activity and hypokinesia due to limited movement inside the spacecraft, structural changes seem to be related to both a higher level of muscle protein degradation enzymes and a decrease in protein synthesis [59]. Extended exposure to microgravity also results in bone loss due to insufficient bone formation compared with bone resorption, and this reduction in bone mass and strength during spaceflight increases the risk of bone fracture, even upon return to Earth [62]. As reported for radiation, similarities have been observed between the adaptive response to microgravity in humans and aging, since both prompt the decline of almost every body system [5]. Though several phenotypic changes resemble those determined by aging under standard gravitational conditions, the magnitude and speed of some processes (bone loss, among others) is far greater (i.e., 1% loss/month) than that which is typically seen during aging. Hence, this is of critical concern when considering the effects of long-duration spaceflight [1]. 

#### 3.1.3. Psychological Stressors

In addition to the aforementioned stressors, several psychological and social issues have been demonstrated to affect the crew during extended separation from society in a closed and confined environment. They may include homesickness and loneliness, apathy, interpersonal stressors, and sexual attraction/tension [63]. A constricted living environment can lead to isolation, loss of spatial capacity, increased anxiety and depression, which can be accompanied by symptoms such as hallucinations, reduced consciousness and poor bodily coordination [64,65]. Additional psychological triggers range from external physical hazards such as space debris and vehicular malfunction to gravitational shifts and outer space radiation [65]. The ability of ionizing radiation to modulate the psycho-emotional status and, specifically, to exert an anxiogenic effect on the central nervous system was shown in rats exposed to doses related to deep-space missions [66]. In addition, diet has a critical role in both the physiological and the psychological health of space travelers. The spacecraft environment, in particular the lack of ultraviolet exposure, increased carbon dioxide levels, the spacesuit atmosphere, etc., can affect nutrition requirements for long-duration missions. Crewmembers may even experience a reduction in their food intake, dubbed “anorexia in space” which may be linked to microgravity, alterations in the circadian rhythm (continuous light environment of space missions) and “menu fatigue”, which not only affects appetite but the proper functioning of the gastrointestinal system [64]. Hence, nutritious and palatable food is necessary not only to meet nutritional requirements and avoid deficiency diseases, but also to keep astronauts psychologically healthy [67]. 

Excessive exposure to noise, mainly due to equipment and crew activities, may represent another stressor compromising well-being as well as sleeping patterns [68]. As a matter of fact, the duration of an astronaut’s sleep is reportedly reduced to around 6 to 6.5 h/day during missions [69]. Psychosomatic disorders (e.g., headaches, fear of illness, gastroenteric problems), consisting of distressing physical symptoms which are not fully explained by a real physical condition, have also been reported from space [64]. Similarly, post-return personality changes and psychiatric problems have been observed in space travelers [70]. Other stress factors that may arise in a multicultural crew are related to language barriers, stereotypes and cultural misunderstandings [67]. Additionally, new psychological stressors could appear in deep-space missions that were not present in missions closer to Earth, and already-known issues could be intensified, leading to stress and problematic behaviors that may interfere with a crew’s productivity and relationships [71]. For example, during a multi-year spaceflight such as that required for a mission to Mars, the crew would be confronted with no access to most of the mitigation strategies currently in place on the ISS, such as real-time communications with family and ground-based mission control and the view of Earth, that contribute to their psychological well-being [48]. Based on the “Earth-out-of-view phenomenon”, humans traveling in outer space might start feeling unconnected to Earth and to family and friends [72]. On the other hand, delays in crew–ground communication (up to 22 min on Mars) would require astronauts to operate more autonomously from mission control [73]. Furthermore, people on a Mars expedition will need to depend on local resources to generate water and fuel for the return home and thus, the psychology of this dependance is an important issue to be considered [70]. In this context, a greater likelihood of withdrawal, territorial behavior and asthenia may occur [74]. The latter is a problematic syndrome quite commonly observed during long-duration missions that produces fatigue, irritability, attention and concentration difficulties, along with heightened perceptual sensitivities, physical weakness, sleep and appetite problems, etc. [64,75].

#### 3.1.4. Additional Risk Factors 

Psychological stress, circadian rhythms, and sleep are key factors strongly connected with one another, as well as with the immune system. This is especially relevant since microbial infection is another challenge facing space travelers. The isolation of opportunistic and pathogenic microorganisms from spacecraft and space stations has been frequently reported [76,77,78] and several studies have demonstrated that spaceflight affects both the immune system (i.e., immune dysregulation) [79] and microbial physiology (i.e., enhanced virulence, biofilm formation and antibiotics resistance) [80], leading to increased risk of disease [81]. Additionally, the proximity of crewmembers to one another in the spacecraft can promote the spread of secondary infections [82]. Due to limited access to medical care during a mission, any possibility of infection should be prevented to ensure the health and safety of the astronauts and to maximize the success rate of the space mission [83].

Crews do not experience the aforementioned stressors independently; therefore, it is important to consider the combined effects of these space environment threats on human physiology, psychology, and performance. These stressful conditions, collectively referred to as “the space exposome” [6], may also exacerbate complex health problems in astronauts embarking on long-duration missions [3]. Space agencies have adopted a number of operational mitigations, direct and indirect countermeasures, whose aim is to lessen the clinical risks related to the physical and psychological stressors associated with space flight. These encompass strict exercise regimes, the use of devices to induce footward fluid shift (i.e., lower body negative pressure, LBNP) [2], pre-flight quarantine to reduce contact with potential pathogens [6], radiation-shielding spacesuits [84], the use of HEPA air filters and in-line water filters in the spacecraft, nutritional supplementation, vaccination, psychological support, etc. [6]. 

While some of the changes induced by exposure to space conditions (e.g., reduction in some motor functions) reportedly disappear shortly or a few months after the end of LEO missions [85], it should be noted that risk assessments and mitigation for lunar visit/habitation, deep-space journey/habitation and planetary missions remain uncertain [86]. If certain medical risks, such as nutrition, infection, psychological impact and even death, appear to be common to all mission profiles, other risks are actually unique to individual scenarios [87]. For instance, risks associated with lunar missions—e.g., lunar surface operations, a lunar outpost, etc.—along with radiation, microgravity and the aforementioned psychological issues, also include exposure to hazardous materials such as rocket fuel, lunar dust (regolith), micrometeorite impact damage, and extremes of temperature [87]. Similarly, perchlorates in the Martian dust would be a concern in terms of contamination of the habitats and of inhalation of harmful particles, posing a great risk to the lung already affected by altered pulmonary deposition induced by microgravity [88]. The optimization of current and novel countermeasures will therefore be critical. 

### 3.2. Effects of Spaceflight Conditions on the Human Microbiome

It is apparent that the space environment imposes several challenges to human physiology. Specific space environment factors, such as microgravity and radiation, are thought to also induce changes in microbiome composition (i.e., dysbiosis) [89], which may alter host–microbe interactions and adversely affect immune function and metabolism, thereby representing a risk to astronaut health, especially during long-term spaceflight missions. 

A list of reviewed microbiome studies pertaining to spaceflight as well as ground-based simulations (conducted through to 2021) is provided in Table 1.

#### 3.2.1. Gut Microbiome

The gut microbiome, often described as the “virtual organ of the human body” [104], will play a crucial and significant role in maintaining astronaut health during space travel, as it does for humans on Earth. High microbiome diversity and richness are generally considered a hallmark of a healthy gut ecosystem; however, there is still no consensus on the actual health-related values [105]. Healthy adult humans characteristically harbor more than 1000 species of bacteria, with *Bacteroidetes* and *Firmicutes* being the dominant phyla [106]. While *Bacteroidetes* (recently renamed as *Bacteroidota* [107]) are connected with immunomodulation and augmented immune reactions through synthesis of cytokines, *Firmicutes* are involved in the metabolism, nutrition, and regulation of hunger and satiety, via short-chain fatty acid (SCFA) synthesis [108]. Exposure to various stressors can change the stability of the gut microbiota, impacting its composition and functions, and increasing the relative abundance of potentially harmful bacteria (e.g., opportunistic pathogens) [109,110,111,112,113]. 

Studies have been conducted to monitor changes in the gut microbiome under real or simulated spaceflight conditions, involving both animal and human subjects. Culture-dependent techniques, substantially used in the past, have been in recent years replaced by high-throughput omics technologies—i.e., 16S ribosomal RNA gene analysis and metagenomic sequencing—which by detecting and measuring also non-cultivable strains have allowed a more comprehensive characterization of the microbiome structure and its biological functions [114]. Despite the advent of new technologies, the number of reports is still limited, and the dynamics of the gut microbiome during space missions are yet to be fully elucidated. 

A recent analysis of fecal samples from mice flown on the ISS for 37 days revealed spaceflight-associated changes in the gut microbiome as compared to the ground controls. These changes consisted of an altered community structure (i.e., an elevated *Firmicutes*-to-*Bacteroides* ratio), higher abundance of bacteria belonging to the order *Clostridiales* and a reduction in the number of *Lactobacillales* (organisms usually considered probiotics), with these changes connected to an altered liver transcriptome [89]. The richness of the microbiome, however, remained unchanged. Similar trends were previously observed in a study reporting the effects of 13-day spaceflight on female mice and were confirmed using a ground-based model of microgravity [92]. The above results are comparable with data collected from Voorhies et al. (2019), which assessed the impact of long-term space travel on the crew microbiome and surrounding ISS environment, and the consequence on human health [98]. A total of nine crewmembers were sampled pre-, during and post-flight, comparing 6-month and 1-year missions. Results indicated that the microbiome composition became more similar between astronauts over the course of the mission, mostly due to a drop in the abundance of a few bacterial taxa [98]. It was revealed that 13 of 17 genera, whose abundance significantly changed in space, were *Firmicutes*, mainly belonging to the order *Clostridiales*. Specifically, the authors reported higher proportions of *Faecalibacterium*, which is known to be a beneficial SCFA producer (i.e., butyrate), but also of genera associated with chronic intestinal inflammation, such as *Parasutterella*. At the same time, it was observed a greater than five-fold reduction in the relative abundance of *Akkermansia*, a genus with anti-inflammatory properties, which according to the authors, could play a role in the moderate increase in the inflammatory immune response observed in the crew during spaceflight. Accordingly, the administration in space of prebiotics or next-generation probiotics, such as *Akkermansia*, has been proposed [115]. Previous sampling campaigns carried out for the Skylab program had also reported a decrease in the diversity of the gastrointestinal community, although the overall microbial count went up following space flight. The data showed an increase in certain pathogenic strains, i.e., *Serratia marcescens* and *Staphylococcus aureus*, the latter of which was found to be transmitted among astronauts, thereby indicating the transmission of pathogens between individuals in the spaceship environment [91]. 

The “Twins Study” has provided the unique chance to evaluate the impact of long-duration flight on the gut microbiome by comparing profiles of an astronaut and his twin, who, by remaining on Earth, served as ground control. The study aimed to monitor various health parameters including changes in ocular, cardiovascular, cognitive and immune functions, as well as cell-specific changes in physiology, transcriptome, proteome, metabolome, epigenome, and telomere length, while controlling for genetics [97]. Although each subject maintained individual microbiome characteristics, more changes were found to occur in the microbial community composition and function during the flight period. In line with what previously observed in other spaceflight experiments [89], a specific increase in the *Firmicutes*-to-*Bacteroidetes* ratio was detected during the 1-year flight period onboard the ISS. Interestingly, this was a transient change, not persisting upon return to Earth, indicating a rebound across the microbial ecosystem of the gastrointestinal tract. Moreover, as anticipated above, microbiome richness composition remained substantially unchanged. 

Alterations in the composition and functionality of the gut microbiome can be induced even by short-term space travel. Liu et al. (2020) reported shifts between dominant genera in the microbiome during space missions of 15 and 35 days that led to increased abundance of *Bacteroides*. By contrast, the probiotic taxa *Lactobacillus* and *Bifidobacterium* appeared reduced, possibly affecting host immune function [81]. Individual specificity was, however, uncompromised. These changes were accompanied by fluctuations in virulence and antibiotic resistance genes and in mobile genetic elements, and by an increase in genes related to biofilm formation [81], which are suggestive of enhanced virulence potential and possibility of infection by opportunistic pathogens or pathobiont of the gut microbiota in space missions [115]. Changes in the intestinal microbiota were also reported in response to increased gravity, a condition experienced by astronauts during specific flight phases such as launch, ascent and descent [58]. A study conducted on mice by Alauzet et al. (2019) revealed disruption of intracaecal microbiota following exposure to hypergravity (3G) for 21 days, which resulted in a decrease in the *Firmicutes*-to-*Bacteroidetes* ratio, however without alteration of mucosal integrity [96]. Of interest, a significant diminution of *Proteobacteria* was observed at 3G, while the opposite was observed for potentially deleterious taxa, such as members of the *Paraprevotella* genus which have been described as being more prevalent in intestinal lumen of patients with colorectal cancer [116]. 

Rearrangements in microbiome composition have also been observed in ground-based analog missions. With the MARS500 study, the temporal dynamics of the gut microbiome of six male crewmembers were monitored over 520 days of isolation within an analog Mars-surface habitat [94]. During the stay in the spacecraft-like habitat, the crewmembers performed realistic activities of a round-trip mission to Mars, including operative work, scientific experiments, exercise and even simulated emergency events, and their access to water and food, whose composition reflected the diet used in the ISS, was limited as in a real space flight [94]. Fecal samples were collected not only during the mission but 10 days before and up until 6 months following the return to normal life, making it the longest controlled human confinement study conducted to date. In the first stage of the mission, an increase in the *Bacteroides*-to-*Firmicutes* ratio was detected, which is consistent with observations from another analog mission, the “Skylab Medical Experiments Altitude Test” carried out in the 1970s in a 56-day confinement environment [117]. Additionally, the study revealed decreased proportions of some SCFA producers, especially *Faecalibacterium prausnitzii* (a butyrate producer), that reached their lowest value at about 1 year of confinement. Not only were increased relative abundance of *Bacteroides* and a decrease in SCFA producers observed in all subjects involved in the study, but these findings paralleled psychological and physiological data that hint at the presence of both mental and physical stress. The authors thereby suggested that changes in this kind in gut microbiota components could be used for the early diagnosis of potential health warnings. MARS500 project data from early (days 7–45) and late (days 420–520) fecal samples were recently reanalyzed using improved 16S rRNA gene amplicon bioinformatics technology [102]. The reanalysis confirmed a significant alteration in relative abundance of the microbiome throughout the period of the study, which included species known to influence inflammation and glucose homeostasis in their host (e.g., *F. prausnitzii*, *Ruminococcus bromii*, *Blautia luti*, *Anaerostipes hadrus*, *Roseburia faecis*, and *Lactobacillus rogosae*) and was consistent with crewmembers’ symptoms. Moreover, a certain level of species overlap could be observed between crewmembers and their habitat: 49 species were shared, representing 49% and 12% of the human and environmental microbiome diversity, respectively. 

Convergence in the microbiota composition of crewmembers (*n* = 3) was also observed in a study monitoring a 105-day analog mission that took place in the Chinese Lunar Palace 1 (LP1) [95]. Results also showed the beneficial influence of the LP1 bioregenerative life-support system (a closed ecosystem integrating efficient higher plant cultivation) dietary structure and a balanced lifestyle (daily diet, living and working activities strictly followed a regular schedule) on the maintenance of a healthy gut microbiota. A high-plant and high-fiber diet resulted in higher microbiome diversity and richness and specifically, a higher abundance of bacteria of the genera *Lachnospira, Faecalibacterium* and *Blautia* of the *Firmicutes* phylum that are known to metabolize dietary polysaccharides and to have anti-inflammatory properties [118]. 

These studies are indicative that modifications in the astronaut gut microbiome occur during spaceflight and analog missions, but the full implications of these findings are yet to be determined in relation to the risks for human health and performance during space travel [119]. Analyses showed that changes to gut microbiome composition are reversible, with at least partial reversal occurring in the order of days to weeks following return to Earth or completion of an analog mission [89,97,98]. However, with extended exploration missions, microbial changes will persist for longer as a consequence of flight duration, due to the limited opportunity of microbial replenishment as compared to individuals on Earth, and this may have long-lasting and serious side-effects, even upon return to Earth [77]. It is also unknown how long after extended spaceflight the microbiome will return to pre-flight levels. 

The combination of space stressors can also impact the gut metabolome, as observed by Casero et al. (2017), where continuous exposure to space-type radiation led to functional shifts in metabolic pathways dominated by microbiome-specific enzymatic reactions [93]. Changes in small-molecule markers of microbial metabolism were also observed in the “Twins Study”, along with particularly low levels of metabolites with anti-inflammatory activity [97]. In this context, looking into the metabolome of the gut microbiota and its modulation as a result of the spaceflight environment may be promising to aid the development of countermeasures that include the use of prebiotics, probiotics and postbiotics to prevent and mitigate pathological effects in astronauts [112].

#### 3.2.2. Skin, Oropharyngeal and Nasal Microbiome

**Figure 2:**
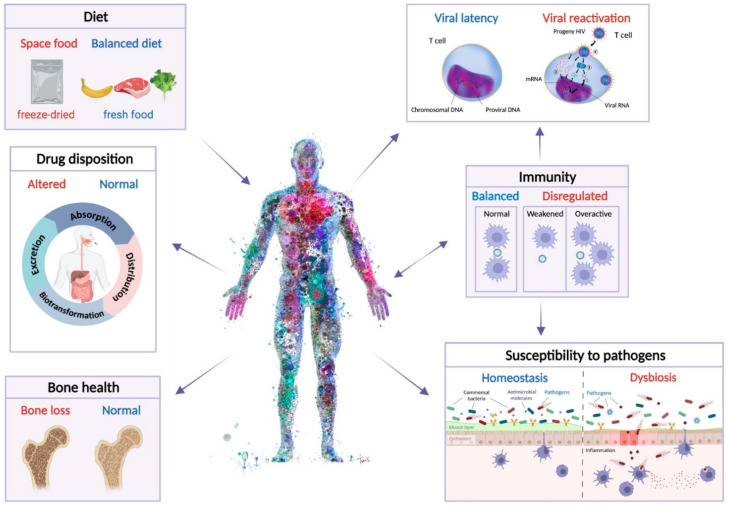
**Impact of spaceflight-induced microbiome alterations on human biology and physiology.** Physiology on Earth (in blue) is compared to physiology in space (in red). Factors that can influence the microbiome—i.e., diet—or that can both influence and be influenced by the microbiome—i.e., immunity—are also shown. Central illustration (human body) “I, virus, the body”, courtesy of Charis Tsevis. Viral latency and reactivation images, courtesy of Alamy Stock Photo. Figure created with BioRender.com (accessed on 18 February 2022).

Microbiome communities residing in the nose and oral cavities and on the skin have also been investigated in relation to spaceflight-induced compositional and functional changes. Located at the entrance of the upper respiratory tract, the nasal cavity and oropharynx serve as the physical barrier to the invasion of pathogens as well as habitats for a large number of commensals and opportunistic pathogens that live in the host as part of the normal microfloa [103]. In the skin, most resident microbes behave as commensal or mutualistic under steady-state conditions and play important roles in the maturation and homeostasis of cutaneous immunity [120]. The disruption of the balance of the microbiota associated with the human respiratory tract and to the skin may result in an increased susceptibility to infection and to the overgrowth of pathogens (Figure 2). On that basis, surveillance of the microbiota structure may be crucial to counteract significant health risks during long-term flight and to guide medical treatment. Moreover, since astronaut skin is the primary source of spacecraft surface contamination, monitoring skin alterations and alterations in the skin-associated microbiome is key to managing astronaut health as well as in the maintenance of space stations, spaceships and spacecraft equipment [121]. 

Early analyses of the microbiome revealed a reduction in the number of nonpathogenic bacteria and an increase in the number of opportunistic pathogens in the nasal flora of astronauts [122]. Similarly, culture-based analyses conducted during the Skylab missions registered noteworthy elevations in counts of anaerobic bacteria, streptococci, Neisseria, lactobacilli and enteric bacilli in the oral microflora, in-flight compared to pre-flight samples from a total of 18 astronauts [90]; however, none of these changes were considered hazardous to astronauts’ health. These findings are consistent with the work conducted by Voorhies et al. (2019), discussed earlier, which showed that the microbiome composition of skin, nose and tongue, such as the gut microbiome, changes in microgravity, and additionally, becomes more similar between astronauts [98]. 

A more recent study used metagenomic sequencing to investigate the microbial profile of mouth, skin, nose, ear, and saliva swabs collected from an astronaut at eight different time points prior to, during and post-spaceflight [99]. While the main objective of the study was to determine the influence of the crew microbiome on the microbial composition of ISS habitable surfaces, it was noted that in saliva samples a flight-dependent decrease in species diversity was observed along with an increase in the relative abundance of *Alloprevotella* [99], a genus associated with dental caries [123]. Interestingly, an increase in the effective number of species was recorded in the samples after returning to Earth. 

In a later study, shotgun metagenomic sequencing and microarrays were applied to characterize the microbial diversity of four astronauts, before, during and following spaceflight on the ISS [100]. The authors reported that astronaut microbiome composition of body swabs and saliva samples changed during spaceflight but went back to normal post-flight. Moreover, these changes were not universal for all four crew members. Interestingly, the relative abundance of the genus *Prevotella* was found to be increased in the saliva samples of two astronauts. The genus consists of several common oral species and increased abundance has been linked to a diseased periodontal state [124]. Additional changes observed in the saliva samples concerned antimicrobial resistance genes: most notably, the elfamycin resistance gene significantly increased in all four astronauts following return to Earth, an aspect which should be considered when administrating antibiotic treatments post spaceflight.

In another recent study, Urbaniak et al. (2020) used 16S rRNA gene amplicon sequencing to monitor spaceflight-induced salivary microbiome changes [101]. Based on the data on microbial composition and diversity, the authors suggested that astronauts’ microbiome can adapt to spaceflight conditions and, moreover, is less recalcitrant to microbiome effects during spaceflight upon re-exposure. Half of the participants involved in the study (i.e., 5) had distinct microbial communities pre-flight, in-flight, and post-flight. Quite interestingly, these subjects were on their first mission, while the other five subjects, who had previously flown to the ISS, did not display microbiome differences. *Streptococcus* was the most abundant organism in the saliva (i.e., 8% of the total organisms detected) and their diversity decreased during spaceflight.

Changes in the skin and nose microbiome were investigated in a study by Voorhies et al. (2019) [98] that aimed at a thorough characterization of the microbiome’s fluctuation during 6- to 12-month space exploration. The forehead and forearm skin microbiota of 9 astronauts appeared to be differentially influenced by the ISS environment: diversity and richness increased or decreased depending upon the individual; however, it was consistent between the two sampled areas [98]. Moreover, a common shift in the microbial composition was observed in all crew members and affected the abundance of *Proteobacteria*, mostly *Gammaproteobacteria* and *Betaproteobacteria*. A concomitant increase in *Firmicutes*, including the genera *Staphylococcus* and *Streptococcus*, was also observed. The authors speculated that decreased levels of *Gammaproteobacteria* may possibly be due to the lack of a “green” natural environment, the constant filtration of air and the alteration of the skin structure during spaceflight [98]. As a reduction in skin *Gammaproteobacteria* has been associated with inflammation and allergy sensitization [125], it is possible that it also plays a role in the occurrence of skin hypersensitivity reactions, rashes, and skin infections, frequently observed in astronauts [126]. In this scenario, skin infections caused by opportunistic pathogens, such as the staphylococcal and streptococcal species, may be facilitated. Fewer spaceflight-dependent changes were found in the nose microbiome, as compared to the skin; however, they concerned the same bacterial genera, whose abundance was found to be modulated in skin (i.e., increase in *Staphylococcus*, *Corynebacterium-1* and *Bifidobacterium*). An elevated relative abundance of clinical pathogenic bacteria such as *Staphylococcus* in the nose has been reported by several studies in association with chronic rhinosinusitis, allergic rhinitis, and asthma [127,128]. Hence, it is consistent with symptoms such as prolonged congestion, rhinitis, and sneezing, which have been reported by astronauts. Nonetheless, other factors could also be playing a role in it. Many of the observed changes in the nose microbiome persisted for at least 2 months after the astronauts returned to Earth. 

Increased abundance of *Staphylococcus* in the nasal cavity has also been reported in a recent study examining temporal characteristics of the oropharyngeal and nasal microbiome during a 180-day ground-based confined experiment in the Controlled Ecological Life Support System (CELSS) [103]. 16S rDNA high-throughput sequencing was used to analyze data from four volunteers at eight time points during confinement and the results showed that the structure of the oropharyngeal and nasal microbiota varied greatly. Individual differences were also observed, with bacterial community structure and diversity changing with time. As with *Staphylococcus* in the nasal cavity, the abundance of *Neisseria* increased over time in the oropharynx. *Staphylococcus* in particular showed the characteristics of inter-individual transfer, suggesting that the microbiota structure and health of the respiratory tract could be affected by living in a closed environment for a long time.

To date, studies have revealed modifications in the skin, oral and nasal microbiome as a consequence of both spaceflight conditions and confinement in ground-based experiments. Changes at the individual level, and specifically an increase in a microbiome’s richness and diversity—i.e., alpha diversity—were reported during spaceflight in studies of the saliva microbiome [100,101], but changes at the populational level were not detected. By contrast, a decrease in alpha diversity in in-flight saliva samples was indicated by an earlier report [99], but this trend was not consistent across all four astronauts involved in the study. Mixed responses were detected in nasal and skin samples [98,103]. Individual differences in the reaction of the skin microbiota to spaceflight may be attributed to the composition of the microbial communities, but also to skin-specific properties such as moisture and pH and/or astronauts’ personal hygiene habits [98]. Concerning the nasal microbiome, changes were observed in relation to both spaceflight [98] and ground-based confined experiments [103], suggesting that the nasal flora of all crewmembers may evolve in the same direction. Microbial transfer between individuals could play a role in it, suggesting that the microbiota structure and health of the respiratory tract could be affected by living in a closed environment for a long time [103]. Aspects including microbial interactions and exchange of microbiota within the crew or with the environment have not yet been fully elucidated and further studies will therefore need to be conducted.

### 3.3. Impact of Spaceflight Conditions on Microbial Physiology and Host-Microbe Interactions

Space microbiology studies have suggested that microgravity is a dominant factor influencing bacterial growth kinetics and cell behavior, and that space radiation may be responsible for increased mutation rates in microbes [129]. Several in vitro studies with bacteria have reported significant in-flight responses that included increased growth rate and cell concentration [130]. Other studies indicated that microgravity is associated with changes in gene expression and virulence factors and can promote antibiotic resistance and elevated transfer rates of genetic material between cells [131,132]. Microgravity-induced changes also include increased membrane integrity and differential secondary metabolite production [133]. 

Decreased susceptibility of bacterial pathogens to antimicrobial agents has been repeatedly observed during space missions (e.g., Cytos 2 experiment, Antibio experiment during the Spacelab D1 mission, Space Shuttle STS-42 mission, etc.) and in ground-based simulations using model organisms such as *Escherichia coli* and *Staphylococcus aureus* [134]. These finding show that in space, bacteria adapt to grow at higher antibiotic concentrations, compared to ground samples [135]. Similarly, resistance to a broad range of antibiotics (e.g., chloramphenicol and cefalotin, persisting for over 110 generations) [136], as well as increased production of the heat-labile enterotoxin [137] and enhanced adherence to mammalian gastrointestinal epithelium [138], have been observed in *E. coli* under simulated microgravity (SMG) conditions.

Moreover, increased virulence and resistance to environmental stress were observed in cultures of the pathogenic bacterium *Salmonella typhimurium* grown under spaceflight conditions, together with increased survival in murine macrophages following oral infection [139]. The mechanisms contributing to this enhanced virulence were the Hfq pathway, which is required for virulence in several bacterial pathogens and considered a global regulator of the microbial response to spaceflight [140], and extracellular matrix accumulation, which is part of biofilm formation. Biofilms protect bacteria from various environmental conditions and increased production of biofilm communities has been frequently observed in bacteria exposed to both simulated and real space conditions [134,141]. In *Candida albicans*, such a feature was found in combination with increased filamentation and increased amphotericin B resistance [142]. In spaceflight-grown bacteria (e.g., the opportunistic pathogen *Pseudomonas aeruginosa*), biofilms tend to show enhanced resistance towards disinfectants, antibiotics and environmental stresses [143]. Still in *P. aeruginosa* and in few other bacterial species, microgravity appears to also stimulate production of signaling molecules (e.g., *N-Acyl homoserine lactone*) able to trigger bacterial communication and to regulate virulence [144].

Considering the effects of spaceflight-dependent alterations on bacterial mono-cultures, the impact of spaceflight on host-associated microbial communities can potentially be more profound. It is known that enhanced virulence of potential pathogens, immune system dysregulation, dysbiosis of the gut microbiome, and disruption of mutualistic interactions, can all be induced by microgravity [79,137,145]; however, the effects of prolonged exposure to microgravity—or various gravitational shifts (i.e., hypo- or hypergavity) that astronauts will face during deep-spaceflight missions—on the complex relationship between host and its microbiome are yet to be fully elucidated. 

Microbe–animal interactions, e.g., mutualistic, pathogenic/parasitic or commensal, and functional networks of microbe–microbe can be equally affected by gravities encountered beyond LEO, with consequences for host health and wellbeing. These interactions rely on a complex system of communication between each member of a microbial community, that influences growth, physiology and metabolism [146]. One such example is horizontal gene transfer (HGT), which is the transfer of genetic material between microorganisms, through either transformation, conjugation or transduction. Research has shown that the human microbiome is a hotspot of HGT [147,148,149], with the rate of HGT between human-associated bacteria 25-fold higher than that among ecologically diverse non-human isolates [150]. HGT is significant as it regulates the exchange of antimicrobial-resistant (AMR) and virulence genes and is the main mechanism driving antibiotic resistance in bacterial communities [151]. Spaceflight conditions reportedly influence gene transfer and enhance HGT. For example, the content of mobile genetic elements appears to be higher in the genome of bacterial isolates grown in space (ISS) than in isolates of the same species from extreme built environments on Earth [152]. In line with this, HGT activity concerning the transfer of AMR genes was found to be more increased in bacteria under simulated microgravity conditions than in 1 G controls [80]. The transfer of these genes from donor (i.e., *Acinetobacter pitti*) to co-cultured recipient strains of *Staphylococcus aureus*, resulted in a phenotypic change, as the recipient strains developed resistance to the antibiotic oxacillin, which they were previously susceptible to [80]. A similar study conducted on the ISS showed increased gene transfer as well, using a phenomenological model. However, plasmid stability was short lived, resulting in overall lower antibiotic resistance compared to ground controls [153]. According to the authors, the reason lies in the fact that processes of segregation and spontaneous elimination of drug resistance genes would prevail over the processes of their cointegration [153]. The exact mechanism influencing enhanced HGT is yet to be determined; nonetheless, it may be related to increased competence or transduction, known to be promoted in bacteria in response to different stressors [154]. Regardless of the mechanisms, the potential impact of the space environment in promoting increased spread of determinants associated with antibiotic resistance and virulence, is of particular concern. Furthermore, risks are not restricted to the astronauts’ microbiome but may as well arise within the microbial flora populating the spacecraft environment (air, surface, water, etc.).

Acquired antibiotic resistance can have serious implications as the efficacy of antibiotic treatment may be diminished during space missions [129]. Although antibiotic resistance may also increase during short-term spaceflights [155], in the context of long-term space missions, strategy and tactics of using antibiotics would have particular relevance. Over-prescribed antibiotic therapy can generally represent an infectious risk factor, potentially resulting in accumulation of antibiotic resistance and pathogenic genes on certain strains due to the process of plasmid mobilization and cointegration [153]. In confined compartments such as spacecrafts, this issue is of even greater concern, not only since microbial communities are a pool for the propagation of antibiotic resistance genes, but also because the transmission of a resistant strain among individuals would be facilitated [79]. Under these conditions, the formation and spread of multidrug-resistant strains of microorganisms and of strains with increased virulence (disease-causing microbes) could be promoted, which, in conjunction with dysbiosis and a possible weakened immune system during spaceflight, carries the risk of increased severity of crewmembers’ infection in long-term space missions [156]. 

More studies will be needed to further our understanding of space environment-driven microbiome changes during long missions, to clarify whether the effects on communities are potentiated or dampened and what the consequences are for the host. In order to obtain a realistic overview of host–microbiome relationships, investigations should not be limited to disease-causing microbes that represent a potential risk to astronauts but shall also include mutualistic and commensal microbes. This can be crucial to learn what drives microbial fitness in the spaceflight environment and how to maintain a healthy symbiotic homeostasis [133].

### 3.4. Influence of the Space Dietary Regime on the Microbiome

Food intake is strictly connected to the composition and the functionality of the microbiome. Research has shown that changes in the diet are followed by rearrangement of the gut flora that can occur within just 24 h of initiation and have secondary effects on host immunologic and metabolic markers [157]. As such, protein consumption positively correlates with overall microbial diversity, with animal protein intake leading to increased abundance of bile-tolerant organisms such as *Bacteroides*, *Alistipes*, and *Bilophila* [158], whereas a diet rich in fibers tends to increase the abundance of bifidobacteria and lactic acid bacteria, known for their anti-inflammatory properties [118]. Along with the type of food, the food’s form (raw or cooked) appears to influence diet-driven host–microbial interactions, with cooking impacting the gut microbiome differently on meat versus plant-based (e.g., tuber) diets [159]. Additionally, multiple compounds with known antimicrobial effects are significantly decreased in cooked food, thus limiting their bioactivity [160]. The close relationship between diet, the gut microbiome, and health suggests that modulations in the diet can have a beneficial or detrimental impact on our health, depending on the relative identity and abundance of constituent bacterial population [161].

It follows that a change in diet such as that faced by astronauts, can also potentially affect the gut microbiome with repercussions on their health [115]. Space food includes a variety of products specifically created for the consumption in outer space and as such, it must meet certain criteria: nutritional properties in line with the crew’s recommended caloric intake, palatability, ease of preparation and storage, etc. [67]. Since water, storage, crew time, and food preparation capability—which does not include cooking—are limited, current space food consists of a narrow choice of shelf-stable, single-serving food products either in their natural form or preserved by dehydration, retort thermostabilization, or irradiation [162]. The variety and amount of fresh fruit and vegetables vary, and their consumption is limited to a few days or weeks. Hence, the ISS food system, for instance, is dominated by meat and meat products [6] and long-term missions will have to rely on bioregenerative life support systems (BLSSs)—e.g., integrating plant cultivation—to introduce a larger variety of food [71].

The optimization of the food system to mitigate negative effects of spaceflight on crewmembers’ health and performance is paramount. A reduction in the astronauts’ caloric intake to 70–80% of the daily requirement [163] occurs at the beginning of the mission due to space motion sickness; however, it can extend well beyond the first days of flight [164], potentially leading to the loss of both fat and lean tissue mass, and to adverse effects on muscle, bone and cardiovascular systems and motor and cognitive functions [115,165]. Moreover, although the reasons are not entirely clear, insulin resistance and glucose intolerance are frequently observed both in short- and longer-term space missions, and in analog missions [166]. A reduced caloric intake like that often experienced by astronauts, may also lead to a restructuring of the gut microbiome similar to that observed in association with very-low-calorie diet (Figure 2) [167]. Accordingly, a low calorie intake and the consequent weight loss may be paralleled by a decrease in bacterial abundance, impaired nutrient absorption, and enrichment in endogenous enteric pathogens (e.g., *Clostridioides difficile*), suggesting that diet-induced shifts in the gut microbiome may influence colonization resistance and thus host physiology. Low-caloric intake may additionally be associated with increased inflammation and oxidative stress, with possible repercussions on the functioning of the immune system [6]. Countermeasures have been developed to provide more balanced diets with increased average caloric intake, optimized to reduce nutrient deficiency and to improve energy supply to lessen the potential downstream dysregulation of the immune system [168]. Despite these efforts, however, diet imbalance during spaceflight remains an issue, as it depends on multiple factors that are not limited to food quality ad palatability but also include cultural habits, alteration of circadian rhythms and in general, the stress associated with the mission [169]. 

Notwithstanding individual differences, microbiome studies carried out in both real and analog missions generally reported rearrangements in the gut microbiome consistent with the higher abundance of bacteria associated with chronic intestinal inflammation and a concomitant reduction in the number of genera with known anti-inflammatory properties [89,94,98]. In some cases, these data correlated with a moderate increase in the inflammatory immune response observed in the crew during spaceflight [98]. Such changes in relative abundance of gut microbial components are possibly the result of multiple factors connected to spaceflight. However, evidence demonstrated the impact of the diet—i.e., a plant- and fiber-based BLSS-dietary structure—on the maintenance of a high gut microbiome diversity enriched with bacteria having anti-inflammatory action during analog missions [95]. Production of SCFAs through gut microbiome-mediated fermentation of non-digestible carbohydrates was shown to contribute, among other things, to the maintenance of metabolic homeostasis [170]. 

Providing the crewmembers with a balanced diet, possibly delivered through BLSSs and rich in fibers, is therefore of great importance to help prevent nutritional imbalances as well as to preserve a healthy gut microbiome. Due to individual differences, personalized nutritional approaches have been suggested [171]. Furthermore, probiotics-based countermeasures via the supplementation of given bacteria strains (e.g., *Akkermansia, Bifidobacterium*, etc.) [98,172,173] or the administration of SCFA-producing next-generation probiotics (*Faecalibacterium*, *Roseburia*, etc.) [174], have additionally been proposed. 

### 3.5. Microbiome and Crewmembers Mental and Physical Health

As we have emphasized throughout the review, the human microbiome contributes to overall health through different routes, including protection against pathogens, maintenance of the immune system, proper intestinal function, and contribution to metabolic functions [79,106,175]. Competitive exclusion of pathogenic bacteria is one of the main functions exerted by the microbiome communities residing in various anatomic sites of the human body. Antagonistic interactions play a pivotal role in determining the composition of a functional antimicrobial barrier, by means of strategies that include production of antibiotics, secretion of digestive enzymes, and quorum sensing [176]. Accordingly, the establishment of new microbes can be prevented through competition for shared nutrients and other resources, with endogenous bacteria [177]. Moreover, microbial communities help strengthen mucosal barrier function and can stimulate epithelial cells to produce antimicrobial peptides and proteins (e.g., bacteriocins), thereby killing pathogenic competitors and preventing translocation [173,178,179]. In view of this, particular concern has been generated by the documented changes in the astronauts’ microbiome [97,98,100,101] and their potential effects on astronaut health and performance, in conjunction with immune system dysregulations and increased risk of pathogenic infections during spaceflight [103,180]—e.g., transient or permanent governance of pathogenic/opportunistic bacterial species, e.g., *Staphylococcus* spp. [181] along with enhanced HGT and decreased susceptibility of pathogens to antimicrobial agents [80]. As a matter of fact, crewmembers do experience adverse medical events of varying severity during spaceflight missions, related to infectious diseases, which include cold sores, skin and urinary tract infections, lymphadenitis and pharyngitis [182]. 

An altered microbiome is not only associated with the onset of infections but with non-infectious diseases as well, such as inflammatory bowel disease [183], systemic metabolic disorder (e.g., type 2 diabetes and obesity) and allergic reactions and sensitivities [184,185]. Changes in the oropharingeal and in the skin microbiome are implicated in the development and progression of caries, gingivitis, tooth decay, as well as endocarditis and heart disease [186,187,188,189], pharyngitis, asthma and pneumonia [190,191,192], acne, atopic dermatitis, psoriasis and chronic wound pathology [193,194]. Moreover, evidence of microbial dysbiosis has been observed in conjunction with various types of cancers [195], including colorectal cancer (CRC) [30,196] and breast cancer [31], among others. The space environment has been shown to alter the tumor microenvironment and promote tumor cell proliferation, transformation and survival [197]. Indeed, US astronauts have an increased incidence of prostate cancer and melanoma, similar to that observed with airline pilots, compared to the general US population [198]. Considering the role that an altered microbiome plays in cancer development and/or progression, microbial disruption during spaceflight, coupled with higher radiation exposure [55], could put astronauts at an even greater risk of developing certain types of cancers with long-duration missions.

In addition to the aforementioned functions of the microbiome in supporting host physiology, research has more recently shed light on the relationship between the gut microbiome and mental health through what is known as the brain–gut–microbiome axis (BGMA) [199]. BGMA signaling has been suggested to be bi-directional, as not only can gut bacteria influence health and the development of emotional behavior, but psychological states can in turn alter gut health [200]. Moreover, the communication appears to occur directly and indirectly via the central and enteric nervous systems and the vagus nerve, through endocrine, neural and immune pathways [115]. Microbiota–gut–brain communication has hitherto mostly been explored in animal models, with human research lagging behind. However, studies have indicated that the gut microbiota can modulate the BGMA via multiple mechanisms, including alterations in microbial composition (i.e., SCFA-producing bacteria seem to be associated with higher mental quality of life indicators [201]) or the potential production of microbial neuroactive metabolites (e.g., SCFAs, neurotransmitters, hormones or neurotoxic metabolites) [202,203]. Preliminary studies have also demonstrated gut microbiota alterations in subjects affected by neurodevelopment disorders such as schizophrenia and autism spectrum disorders [204,205]. Furthermore, perturbations of the BGMA have been found in association with stress-related gastrointestinal disorders [206], as well as increased anxiety [207], depression [208], Parkinson’s disease [209] and decreased cognitive abilities [203], suggesting that the microbiota may contribute to shaping cognitive networks encompassing emotional and social domains [205]. Of interest, altered BGMA and microbiome have also been found in conjunction with disrupted sleep physiology, with microbiome diversity being positively correlated with increased sleep efficiency and total sleep time [200]. Cognitive or behavioral conditions reported during spaceflight include reduced sleep quality and increased anxiety and depression, which can be accompanied by the impairment of psychomotor functions and neurocognitive performance [210,211]. Occurring as a result of several mission-related environmental (e.g., radiation, microgravity, excessive exposure to noise and light) and psychosocial stressors (e.g., isolation, homesickness), these symptoms represent a threat to the success of space missions, as they greatly affect astronaut wellbeing [212]. In light of the bidirectional interactions between the gut microbiome and the brain [213], based on which the microbiome can influence cognition and emotion, it can be assumed that strategies aimed at maintaining a healthy microbiome might also be helpful in mitigating unwanted neurobehavioral effects [115]. In line with this, research shows that successful treatment of anxiety symptoms can be achieved by regulation of intestinal microbiota by means of both probiotic and non-probiotic (e.g., regulating diet, supplementation of short-chain fructooligosaccharides scFOS) interventions [214]. This aspect is of particular significance as it underlines the far-reaching impact of the gut microbiome and offers new perspectives regarding the understanding and at the same time the mitigation of psychological stressors (external or self-imposed) that may arise during spaceflight.

Although microbiome changes generally seem to rebound after returning to Earth [97], the entity and persistence of alterations induced by longer space missions (e.g., deep-space missions) and the associated risk of increased severity of infection, disease onset, and mental health impairments, are yet to be fully assessed. In the long run, compositional changes in the gut flora might even predispose astronauts to more prolonged-development diseases such as IBS, autoimmunity and even cancer [182]. The risks associated with these factors should therefore be explored in more depth.

## 4. Spaceflight, Microbiome and Immunity 

Understanding the combined effects of spaceflight on the immune system is significant for the health and safety of crewmembers and is an important area of interest as long-term exploration flights become more common. The intense conditions that astronauts encounter in space, including radiation exposure, microgravity, changes in diet, disruption of circadian rhythm and stress, all have an effect on the immune system (Figure 2) [6,215,216,217,218,219,220]. A dysregulated immune response has been well-established during spaceflight, with many changes to immune cell parameters, such as in the distribution, function and proliferation of leukocytes [215,216,221,222,223,224,225,226,227,228,229], cytokine profiles [97,217,219,221,222,229,230,231,232,233] and neutrophil [221,234], monocyte [232,235] and NK cell function [236,237,238]. As discussed earlier, microbial physiology changes as a result of spaceflight, with one such change being increased virulence in some pathogens grown in space [139,144,239,240], which is problematic considering the dysregulated immune responses astronauts experience during spaceflight [6,97]. However, the observation that a vaccine administered in space was able to stimulate an appropriate immune response is promising for long-term missions [97]. The effect of gut microbiome dysbiosis is an essential factor to consider when reflecting on the immunity of astronauts as the microbiome has essential roles in the development and function of both the innate immune system—including regulating neutrophils [241], and macrophages [242,243], and the adaptive immune system—including influence on the function and repertoire of B cells [244], the induction of intestinal IgA [245], the differentiation of Tfh cells [246,247], and transition of antigen-activated CD8 T cells into memory cells [248]. Although there are variations in the results due to different circumstances such as spaceflight duration, sample retrieval and experimental protocols, overall, the generation, function or proportion of immune cells are affected during spaceflight, which disrupts the homeostasis required for an appropriate immune response [97,222,228]. 

Dysbiosis of the gut microbiome has been associated with a dysregulated immune system, where changes in T cell regulation and cytokine secretion are observed [249,250,251,252]. The gut microbiome has a significant role in differentiating naive CD4+ T cells, which defend against extracellular pathogens and suppress the immune system when a response is not required. Microbes such as *Bacteroides fragilis*, segmented filamentous bacteria and *Clostridia* can differentially induce the development of T_H_1, T_H_17 and T_reg_ cells, respectively [19,250,253,254,255]. T cell function is affected in space, but the response differs depending on the flight duration, as short-term missions increase T cell function and long-term missions cause T cell function to decrease upon landing [229]. Cytokine production profiles, such as IFN𝛾, IL-17 and IL-10, are also affected during spaceflight [222,228]. These are cytokines that are secreted from immune cells, which are regulated by the gut microbiome [249,256,257]. Additionally, astronauts experience spaceflight-related reductions in certain SCFA-producing bacteria in their gut microbiome, such as *Pseudobutyrivibrio* and *Akkermansia* [98], which may be a result of the imposed conditions of flight, such as the decrease in dietary fiber in the astronaut diet [6,258]. SCFAs produced by the gut microbiota are important in immune system regulation, as they have a role in CD4+ and CD8+ T cell function, generation and cytokine secretion [259,260]. Butyrate, for example, has been identified in reducing gastrointestinal inflammation through the induction of IL-10, inhibiting the secretion of pro-inflammatory cytokines, and regulating innate immune cells and T_reg_ cells [261,262,263,264]. Although many other factors have been implicated in the dysregulation of the immune system in space, the microbiome’s effect cannot be discounted.

Many astronauts experience uncharacteristic allergies and skin rashes during spaceflight [182], with some requiring antihistamines or steroids to manage these reactions [265]. These hypersensitivities may result from a T_H_2 shift in the immune system, which has been observed to occur in spaceflight [229]. Significantly higher IL-10 levels than IFN𝛾, which suggest a shift towards a T_H_2 response, were also observed among astronauts upon landing [229]. Skin reactions in space could be associated with changes to the skin microbiome since decreases in Gram-negative *Proteobacteria*, which includes *Acinetobacter*, were documented in astronauts [98]. *Acinetobacter* is a commensal skin microbe that can help maintain homeostasis by reducing inflammation, regulating the balance between T_H_1 and T_H_2 cells, and inducing IL-10 production [266,267]. The relative proportions of *Bacteroidetes*, *Actinobacteria* and *Firmicutes*, in particular staphylococcal and streptococcal species, increase in the skin microbiome during space flight [98]. An increase in *Staphylococcus aureus* colonization has been seen in patients with atopic dermatitis on Earth [268,269]. These alterations to the skin microbiome may contribute to the overactive immune responses encountered in space and may contribute to conditions such as erythema, psoriasis, various types of dermatitis and delayed wound healing, which represent frequent skin problems during space missions [126,265,270,271]. 

Increased and persistent reactivation of Epstein–Barr virus (EBV), varicella-zoster virus, herpes-simplex-1, and cytomegalovirus, four naturally occurring latent herpesviruses, have been reported by astronauts during both short- (10–16 days) and long (60–180 days)-duration missions, with viral titers and shedding increased with the length of the mission [272,273]. While a robust and competent immune system is necessary to maintain latency, as observed in spaceflight [231,274] and space analog studies [275,276], a dysbiotic microbiome could also be a contributing factor (Figure 2). This could be through changes in microbiome-immune modulation, or through changes in bacterial–viral interactions. In the case of the latter, metabolites produced from the oral microbiome were shown to influence viral reactivation from latent human immunodeficiency virus, Kaposi’s sarcoma herpesvirus, and EBV, by activating viral promotors or causing epigenetic modifications of the viral genome [277,278,279,280]. Correlation analyses between the salivary microbiome and EBV titers, in astronaut saliva, revealed a strong positive correlation (suggestive of promotion) with *Gracilibacteria* and *Abiotrophia* and a negative correlation (suggestive of protection) with Oribacterium, *Veillonella*, and *Haemophilus* [101]. *Veillonella* is one of the main hydrogen sulfide (H_2_S) producers in the oral cavity [281], a chemical that is also produced by the intestinal microbiome [282]. While H_2_S displays antiviral activity against pathogenic RNA viruses [283,284], it is also regarded as an endogenous regulator of both the innate and adaptive immune arms [285]. Research has shown that therapeutically administered doses of H_2_S can improve the function of various immune cells and protect them against dysfunction from various stressors (reviewed in [285]). Studies are currently limited regarding the role of the microbiome in latent viral reactivation, especially as it pertains to spaceflight. However, this is a topic that warrants further investigation to help reduce the risks and complications of viral infections in astronauts during long-duration missions. While it is important to note that many cases on the ISS are asymptomatic [286], with a lower incidence of reactivation in recent years attributed to better biomedical countermeasures [287], infections that do arise, under certain circumstances, can lead to shingles, mononucleosis, various types of cancers and different inflammatory diseases such as myocarditis and pancreatitis, all of which will be hard to treat in outer space. 

Many immune responses and resulting medical issues encountered by astronauts during spaceflight could be linked to abnormal microbiomes, and further studies should be conducted to gain insight into the mechanisms of these microbiomes in human health and immunity. 

## 5. The Impact of the Built Environment on the Astronaut Microbiome

The microbiome of the built environment is the collective of microbial inhabitants in human-constructed environments [288]. The indoor microbiome is infrastructurally unique and differs between hospitals, offices, classrooms, and homes influenced by variations in material design, ventilation, temperature, humidity, pressure, and occupants [289,290,291,292,293]. Humans leave behind a microbial footprint through shedding, exhalation, and waste, accounting for approximately 40% of the microbes found in buildings [294,295]. It has been shown that less urbanized and more confined environments with reduced outside contact are even more overshadowed by human-associated microbes [296]. The ISS is one such confined environment, a unique habitat where the only exchange with the “outside” comes from the turnover in crew members, cargo capsules and supplies, with crew members being the main source of the ISS built environment microbial community [98,99,297,298]. Of the most abundant microbes catalogued on the ISS are those pertaining to skin, respiratory and gastrointestinal tracts [98,99,299]. These include *Staphylococcus*, *Propionibacterium*, *Actinobacterium*, *Enterobacterium*, *Corynebacterium*, *Streptococcus*, *Acinetobacter*, and *Pantoea*, along with various bacteria belonging to the phylum Firmicutes [78,98,297,299,300]. The ISS microbiome has also been observed to change over time in accordance with flight and exchange of crewmembers suggesting that temporal changes in the built environment may be due to different occupants on board [297]. Earth-based studies in the inflatable lunar/Mars habitat mimicked this trend where microbial communities during complete vacancy at day 0 differed from those seen at day 30 post human occupancy [301]. Although there is ample evidence that the human microbiome can influence the built environment, microbial transfer is not unidirectional. Early microbial studies in Russian astronauts aboard the Salyut and Mir orbital stations, revealed an interchange of gut microbiota between crew members [122,302]. With new metagenomic technologies, a direct transmission between ISS surfaces and the astronaut microbiome has been observed, through either single-nucleotide polymorphism, haplotype matching and/or genomic read recruiting [303,304]. This two-way microbial transfer between the ISS environment and the astronaut suggests ISS occupants can not only help build the microbiome of the ISS, but also uptake it as their own. 

Microbial transfer between astronaut and the ISS environment, as well as astronaut to astronaut (via surfaces) can be hazardous due to the altered immunity astronauts encounter during spaceflight, increasing their susceptibility to infection from opportunistic pathogens (Figure 2) [156,235,236,305,306,307]. Evidence of opportunistic pathogens identified on ISS surfaces include cultivable populations of *Staphylococcus aureus*, *Staphylococcus hominis*, *Staphylococcus haemolyticus*, *Platanthera conspicua*, *Acinetobacter pittii*, *Klebsiella quasipneumoniae*, and *Aspergillus fumigatus* [297]. Although the ISS and astronauts are strictly monitored to prevent risks from pathogenic infection, conjunctivitis, acute upper respiratory tract, and urinary tract infections have been reported by crew members of the ISS [308,309]. Microbial virulence and antimicrobial resistance of these opportunistic pathogens could be further increased by the stressors of space, making infection not only more likely, but possibly harder to treat [217,310,311,312,313]. For example, *Staphylococcus epidermidis* grown in space acquired mutations in the *rpoB* gene, heightening its resistance to rifampcin [314,315] and *Aspergillus fumigatus* isolated from the ISS was significantly more lethal to neutrophil-deficient zebrafish compared to Earth-based clinical isolates [240]. 

Building material can affect microbiome diversity and pathogenesis as well. Materials with higher hygroscopicity and porosity tend to have higher microbiome diversity due to moisture accumulation and environmental protection [316,317,318,319]. A submerged analog habitat that simulates ISS confinement and pressure found that different niches between particle board surfaces (LDP) and glass/metal surfaces selected for different viable microbial communities, with microbes found on LDP surfaces having higher abundance of antimicrobial and virulence associated genes. This suggests that material type can not only affect microbial diversity, but also pathogenicity [318]. It has been hypothesized that increased virulence and AMR resistance in confined environments, with low microbial diversity, are a result of adaptations that help bacteria and fungi survive in these restricted conditions [320,321,322,323]. These genomic and metabolic changes that occur in confined environments could explain the many novel species that have been identified in various confined habitats [324,325,326,327,328]. Efforts toward design of spacecraft materials to mitigate pathogenic growth would benefit from the prevention of infection rather than relying on treatment after infection, with limited medical resources. Analysis of other highly controlled environments such as spacecraft assembly clean rooms and intensive care units revealed that microbes in controlled environments rely more on nitrogen, carbohydrate, and heightened drug metabolisms versus in uncontrolled environments where microbes depend more on oxygen and amino acid metabolisms [329,330]. Understanding how microbes adapt to utilize different resources in a controlled built environment can help provide insight in future spacecraft design.

Another consideration for future spacecraft design is the risk of biofilm formation which can affect astronaut health and spacecraft integrity. Biofilms are associated with a range of disease including cystic fibrosis, osteomyelitis, prostatitis, rhinosinusitis, otitis media, urinary tract infection, endocarditis, periodontitis, and infectious kidney stones [331,332] and are responsible for 80% of chronic and recurrent infections [331,333,334]. Biofilms also induce corrosion, lead to mechanical blockages, and decrease the effectiveness of heat transfer on ISS equipment [335,336,337,338,339], putting spacecraft integrity at risk and posing an indirect safety hazard to the crew. Biofilm forming microbes such as *Acinetobacter*, *Sphingomonas*, *Bacillus*, *Burkholderia*, *Corynebacterium*, *Klebsiella* and fungi *Penicillium*, *Aspergillus*, *Cryptococcus*, *Rhodotorula* have been found on the ISS [297], though it is important to note that almost all species of bacteria can form biofilms under certain conditions; hence, many more biofilm formers could be present on the ISS. 

The astronaut microbiota heavily contributes to the built environment of the ISS. Once transferred from host to environment, stressors such as microgravity, radiation, and confinement can alter pathogenicity, making it an infection risk for crew members should it be transferred to them from the environment. It is therefore vital to continually monitor microbial pathogenesis on the ISS to avoid crew member infections and continue studying the built environment for optimization for future space travel.

## 6. Microbiome and Bone Health 

Astronaut bone loss during space flight has been an unresolved medical concern since the 1970s. Pre- and post-flight measurements of bone density in astronauts quantified with absorptiometry and quantitative computed tomography have shown an overall bone loss rate of 1–1.5% per month with areas of the lumbar spine, pelvis, and lower limbs contributing most heavily to the decline in bone density (Figure 2) [340,341,342,343,344,345]. Measurements of volumetric bone mineral density in astronaut tibias after spaceflight showed a 5-percentile reduction that is comparable to average bone loss occurring in men over twenty years and 6 times faster than the accelerated bone loss that is often seen in menopausal women [346]. These findings have made bone loss treatment and prevention a high priority for astronaut health and safety. 

Bones undergo modeling during development and remodeling in later stages of life to form, replace, and remove bone [347]. Osteoblasts are derived from stromal cells of the bone marrow and are responsible for bone formation [348]. In contrast, osteoclasts that derive from the hematopoietic stem cells in the bone marrow, resorb bone during remodeling, making the equilibrium between the bone forming osteoblast and bone resorbing osteoclast vital to overall bone homeostasis [349,350]. Bone formation and resorption can be biochemically measured in the urine through quantification of proteins produced during formation, such as bone-specific alkaline phosphatase and osteocalcin, as well as peptides released during matrix degradation, such as hydoxyproline, collagen type I, pyridinoline, and deoxypyridinoline [345,351,352]. These biochemical assays have been conducted on astronaut urine before, during, and after spaceflight to reveal that bone resorption markers heavily increase during spaceflight with only a slow increase in bone formation markers [345,353,354,355,356,357,358,359,360,361,362]. Post-flight measurements showed exponential decreases in bone resorption markers, but only a linear increase in bone formation markers, keeping bone homeostasis out of equilibrium during and post flight [345,357,358,359,362,363]. Furthermore, trabecular bone, the sponge-like structure at the epiphyses of long bones involved in metabolic processes associated with bone turnover, was also reduced post-flight [364,365,366]. Compromised trabeculae can irreversibly damage bone structure altogether [367,368]. One hypothesis contributing to region-specific bone loss during spaceflight is the changes in mechanical loading induced by microgravity [369,370]. High mechanical loading zones are reduced to low mechanical loading in microgravity and may therefore also reduce bone for the lack of necessity in that region. Extensive resistive exercise regimes have been employed in astronauts to stimulate mechanical loading to these areas that have shown to reduce bone loss, but do not ameliorate the bone loss process [342,345,365,371,372,373]. It has also been pointed out by Stanichuk et al. that bone density changes were also found in areas of neutral mechanical loading such as the skull, suggesting that microgravity-induced changes in mechanical loading may not be the sole contributor to bone changes during spaceflight [345]. 

With increased understanding of the influence of the microbiome to overall health, many studies have revealed that the gut microbiome can also specifically influence bone health. Proteins and SCFAs produced by the gut microbiome have been shown to promote bone formation [374]. Butyrate, a short-chain fatty acid produce by *Lactobacillus* of the gut microbiome, promotes bone formation through T cell signaling inducing differentiation of osteoblasts in the bone marrow [375,376,377,378]. The gut microbiome is also a rich source of vitamin K2, which is required for the activation of osteocalcin, a protein produced by osteoblasts during bone formation [379,380]. Antibiotic-induced gut microbiome dysbiosis dampens vitamin K2 synthesis and is associated with decreases in osteocalcin and bone strength [381]. Bone loss in models that promote bone resorption can be reduced through dietary supplementation of beneficial bacteria, such as *Lactobacillus reuteri* which protects against bone resorption in estrogen-deficient mice [382,383] and trabecular bone loss during antibiotic dysbiosis in mice [384,385]. 

Gut microbiome dysbiosis has been linked to bone disease in humans with osteoporosis and osteopenia, where diseased patients showed higher microbiome diversity than their healthy counterparts, and the severity of bone loss is positively correlated with higher microbiome diversity [386,387]. Gut microbiome dysbiosis can lead to inflammation followed by intestinal permeability that allows the gut microbiome to enter circulation [388,389]. Microbes in circulation stimulate an immune response from immune cells that recognize lipopolysaccharides using Toll-like receptors. Once recognized, the immune cells then activate to produce cytokines that promote maturation of T_H_17 cells in the bone marrow which then stimulate osteoclastogenesis and bone resorption [390,391,392]. As we have mentioned, the stressors of spaceflight can stimulate astronaut microbiome dysbiosis [110,111,217,393,394]. One such change during spaceflight is the decrease in genera with anti-inflammatory properties in the gut microbiome and the increase in *Parasutterella*, known to be associated with chronic inflammation [98,115]. These microbial changes may be increasing intestinal inflammation, which signals the promotion of bone resorption. Additionally, it is worth noting that astronaut gut microbiomes have been reported to have an increased abundance in *Firmicutes* [81], a similar phenomenon seen in the gut microbiomes of patients with osteoporosis [374,387]. There is evidence of both astronaut bone loss and microbiome dysbiosis during spaceflight; however, research is lacking on a possible association between the two. Additional investigation on this relationship could provide easier methods of inflight bone loss treatment and prevention through dietary pre and probiotic supplementation. Combining microbiome symbiosis with exercise regimes that maintain mechanical loading may help diminish bone loss during space travel.

## 7. Gender Differences 

As space exploration expands to include more long-term missions, the health and safety of both male and female astronauts are important factors to consider. As there are many differences between the sexes, including the composition of their microbiomes, making sure that these differences are identified and evaluated is critical for understanding the impact spaceflight has on crewmembers. Microbiome diversity and composition diverge at similar ages after puberty in males and females, and high testosterone or estradiol levels result in a more diverse gut microbiome [395,396,397]. Studies have shown that microbes in the intestinal tract can impact sex hormone levels, and sex hormones have a role in shaping the gut microbiome composition [396,397,398,399,400,401,402,403]. This bi-directional relationship contributes to the gender-specific differences observed in disease, such as a greater occurrence of CaOx kidney stones [404,405,406,407,408] and earlier onset of cardiovascular disease in males [409], and postmenopausal osteoporosis [410,411] and IBS [412,413] in females. This relationship has also been implicated in ovarian cancer [414,415] and polycystic ovary syndrome [416,417]. Sex hormones have been shown to affect the immune system through interactions with the gut microbiome, including influencing the gut barrier permeability and interacting with immune cells [418,419]. Studies show that there are sex-specific differences in the immune system, which are independent of the gut microbiota and are already present in germ-free mice, suggesting that the immune system can select a gender-specific gut microbiome conformation, which also plays a part in the differential influence on the immune system observed across genders [420]. These gender-specific differences in immunity result in males being more susceptible to infections [421], and females being more susceptible to autoimmune disorders [422]. 

The implications of microbial dysbiosis on astronaut health can be better evaluated when the gender-specific distinctions between male and female microbiomes are considered. Astronauts’ microbiomes are exposed to and influenced by many intense factors in space, and there is evidence that these factors have a gender bias. Astronauts are exposed to cosmic radiation, which has been observed to alter the gut microbiome composition [423]. Cui et al. conducted animal studies with mice to show that the effect of radiation toxicity is more prevalent on female gut microbiomes and that gender-matched fecal microbiota transplantation was most successful in reversing these effects [424]. Females are also more vulnerable to radiation-induced cancer than males, especially radiation-induced breast, lung, thyroid and ovarian cancer [425,426]. Looking more closely at the microbiomes of Chernobyl victims or nuclear power plant workers may be an area of interest to further investigate the long-term effects of radiation [427,428]. There are also extensive lifestyle changes during spaceflight, which include alterations to circadian rhythms and modifications to the diet. Changes in sleep patterns have been observed to have an influence on the gut microbiome, leading to a higher risk of breast cancer in females [429,430,431,432]. Diet has a sex-specific effect on the microbiome, where variations in changes to the microbiome composition have been observed in the presence of different diets and prebiotics [398,433,434,435]. Dietary fibers can affect estrogen levels [436], whose link with the microbiome has been recognized, and high-fat high-sugar diets can affect bile acid production differently across genders, which also has been shown to influence the gut microbiome [398]. 

Astronauts experience high levels of stress in space due to a multitude of factors, including isolation, resulting in higher levels of cortisol and catecholamines [437]. Increased levels of stress-induced cortisol can trigger an inflammation response, disrupt the intestinal barrier and alter microbial composition [438,439,440]. As there are sex-specific differences between the way males and females regulate their stress response, the impact on the microbiome varies [441,442]. Studies examining the effect of isolation on prairie voles showed gender-specific changes in the gut microbiome composition [443]. Dietary supplementation of DHA reduced stress and changed the microbiome composition in socially isolated male mice, but not in female mice, further suggesting the impact of sex-specific stress responses on the microbiome [444]. Cortisol can also negatively impact the vaginal microbiome by inhibiting the glycogen deposition, which can lead to genitourinary tract infections [445]. Therefore, the associated stress of spaceflight has a different impact on male and female microbiomes, which may contribute to the gender-specific associated health risks of space travel. 

The disproportion of men compared to women who have flown to space introduces a bias to the data collected from astronauts and the available evidence limits the conclusions that can be made on the impacts of space exploration on female health. Recognizing gender-specific differences in the microbiome response to the extreme factors of space will allow for better and more personalized countermeasures and medical care to help preserve the homeostasis of the microbiome and as a result, the health of astronauts. 

## 8. Pharmogenetics, Spaceflight and the Microbiome 

Even with countermeasures and preventions put in place to keep astronauts safe during long-duration missions, the extended time in space with prolonged exposure to radiation, weightlessness, and other stressors on the body will inevitably lead to medical issues that arise during flight. These ailments will need to be addressed by pharmaceutical intervention with even more robustness and rigor than in low-earth orbit, due to the inability for emergency evacuation and limited non-medicinal interventions. An excellent review by Blue et al. discusses the challenges and current understanding for NASA in supplying a pharmacy for exploration spaceflight [446]. 

Medication has been used for decades by astronauts during spaceflight to help relieve symptoms such as headaches and muscle/joint pain [447], or for more serious issues, such as treating jugular venous thrombosis [448]. Medical toolkits onboard the ISS now contain about 190 different frequently used pharmaceuticals. However, observational studies and anecdotal reports from crew members indicate medications to be “not effective” or “less effective” at managing their complaints (Figure 2) [446,449,450]. More formal studies support these reports and suggest altered drug disposition in space compared to Earth, which include differences in efficacy, absorption, drug elimination, pro-drug activation and build-up of toxic metabolites [446,447]. 

While human physiological changes [451,452] and reduced drug self-life [453,454] during spaceflight can alter drug disposition in space, the gut microbiome can also contribute to differential drug efficacy and safety, by enzymatically transforming drug structure and altering drug bioavailability, bioactivity, or toxicity [455,456]. For example, the common human gut bacterium, *Eggerthella lenta*, inactivates the cardiac drug digoxin, used to treat heart failure and arrythmia, via a two-gene ‘cardiac glycoside reductase’ (*cgr*) operon, which is conserved and widespread within the human-associated gut microbiome [457]. It has also been shown that co-culture of *E. lenta* with the fecal microbiome increased *cgr* expression, thereby enhancing digoxin metabolism and inactivation [458], reminding us of the interconnectedness of microbial species within a community and its impact on the host. Of interest, dietary protein was able to reduce digoxin metabolism [458], thereby maintaining its efficacy. This has important implications for the crew as a carefully considered “microbiome diet” could help ameliorate any negative effects that may be imposed by a dynamic and changing microbiome caused by spaceflight. 

The gut microbiome can also promote drug activation, which was first discovered in 1937, with the antibiotic prontosil, which required bacterial azoreductases in the gut to cleave the drug into its active form [459]. Since then, other prodrugs have been developed, such as sulfasalazine, balsalazide, and olsalazine, used to treat ulcerative colitis, which rely on colonic bacteria for activation [460]. Unfortunately, microbial-mediated drug metabolism can also lead to toxic side effects, with the most notable example being that of sorivudine, an antiviral agent, which led to the death of 18 people in Japan, and which was withdrawn from the market only weeks after being released [461]. This example stresses the importance of incorporating pharmacomicrobiomics (the study of microbe–drug interactions) [462] when making policies and decisions for planned and future missions. To date, over 270 drugs have been recognized as being susceptible to gut microbiome metabolism, leading to inactive, active or toxic forms [461]. Some that may be of relevance to spaceflight currently or in the future include acetaminophen—used to treat mild to moderate pain and reduce fever [463]; ranitidine and nizatidine—antacids used to treat and prevent stomach ulcers and acid reflux [464]; loperamide—used to treat acute diarrhea [465]; metronidazole—an antibiotic [466]; and methotrexate to treat breast, bone and lung cancer, along with rheumatoid arthritis [467].

There is still a great deal of work that needs to be carried out to understand the host–microbiome–drug response and how it affects each individual astronaut in flight and on the ground. Understanding how the astronaut microbiome composition, its collective genes, the expression of those genes and the metabolites they produce change during spaceflight and post-flight will undoubtedly help prevent serious side effects from microbe–drug interactions, but also make a positive impact on an astronaut’s response to a given drug. Opportunities for drug substitutions could be available, as multiple drugs within a drug class can be differentially affected by the gut microbiome. For example, while the H2 antagonists ranitidine and nizatidine were quickly metabolized and negatively affected by colonic bacteria, cimetidine and famotidine (also H2 antagonists) were not [464]. In human bedrest analog studies, used to mimic microgravity, on Earth, the pharmacokinetics of amoxicillin was effected [468], but not of penicillin [469,470]. Knowledge of this kind can help us to make more informed decisions of which drugs should be included in the medical toolkit for flight. 

## 9. Recommendations

Maintaining astronaut health and performance is necessary to ensure successful long-duration missions beyond low Earth orbit. With NASA’s long-term plans to include crewed missions to the Moon and Mars, incorporating microbiome data into planning and policies will help astronauts complete these challenging missions and preserve their long-term health. In this section, we provide recommendations on how best to incorporate microbiome research when designing and planning for the next milestone in space exploration. We recommend the following:

- Personalized microbiome monitoring plus personalized countermeasures to strengthen microbiome resilience to deep-space exploration.
- Inclusion of more women astronauts in space biology studies to determine gender-specific effects of space travel.
- Investigations that include a systems biology approach to obtain a comprehensive overview of gene expression and metabolic networks, e.g., the metabolites produced from microbiome and host.
- Many gaps in our understanding exist about host–microbe interactions and how they are essential to human health and wellbeing. It is thus crucial that research be prioritized to assess what are the key beneficial interactions and associated molecular processes that contribute to maintaining function.
- Comprehensive analysis of the impact of space conditions on microbial communities that includes the study of both pathogenic and beneficial microbes and their mutualistic interactions. Though pathogenic microbes represent a potential risk to astronauts, it is essential to have an understanding of mutualistic microbes to learn what drives microbial fitness in the spaceflight environment and how to maintain a healthy homeostasis between humans and their microbiome. Achieving a better understanding of the interplay of changes in microbiome composition and their impact on the astronauts can help in developing prevention or countermeasures.
- There is a need to evaluate the long-term effects of microgravity (or diverse gravity) on the microbiome. So far, studies have been carried out for a maximum of 1 year. Longer studies on the same human subjects are not possible. The development of computational models for simulation and analysis could represent an alternative approach.
- More directed studies regarding probiotics and prebiotics in the astronaut diet, to modulate and balance the microbiome and aid in reducing inflammation, bone loss and other impacts of spaceflight.
- Promote research on built environment material design such as using natural antimicrobial materials or treatment of surfaces with specialized coatings. These can help reduce bacterial load, biofilm formation, HGT transfer and prevent changes in bacterial physiology that could be detrimental to astronaut health and spacecraft integrity.

## 10. Conclusions

By the end of the decade, NASA aims to establish a sustainable habitat on the Moon, followed by the next ambitious plan of human occupation on Mars. NASA, along with other space agencies, government, academia, and industry are striving to address the challenges of living in space for long periods of time. Missions to the Moon would be 1000× farther from the Earth than the International Space Station, and a mission to Mars would last at least 6 months. The 20 years of human studies conducted on the ISS have provided invaluable knowledge of how the human body adapts to the space environment, but more work is needed to understand how the human body will function and adapt to space conditions beyond LEO. One such adaptation is the human microbiome, and as discussed in this review, this plays a significant role in modulating health and disease. We have demonstrated the importance of a balanced microbiome to help maintain astronaut health in orbit and have discussed adverse events experienced by the crew during missions of varying lengths and how the microbiome (either balanced or in dysbiosis) ties into those medical events. Moreover, we have examined less well-established links between spaceflight, bone loss and the microbiome and emphasized the importance of taking into account gender differences when designing appropriate countermeasures for short- and long-term missions. In addition, we have also examined the role that the microbiome can play in altering the effectiveness of pharmaceuticals that are part of the astronaut medical toolkit in space, and the consequence of this for long-term missions. As we move forward with long-term space travel and human habitation beyond LEO, more studies will be needed that explore the astronaut microbiome, the factors governing its stability or disruption and its interaction with the host and the spacecraft environment. We hope that the literature presented in this review and the recommendations provided will help in future study design, technology and product development, and policies that center around the human microbiome, as we propel human exploration beyond anything we have seen so far. 

## Figures and Tables

**Table 1:** Effects of spaceflight, postflight and ground-based analog missions on the host microbiome.

Experimental Conditions	Sample Type	In-Flight Changes	Post-Flight Changes	Methodology	References
Sampling campaigns carried out for the Skylab program on a total of 18 crewmembers. Research included pre-flight and post-flight monitoring.	Gingival sulcus, dental plaque, and saliva.	Increase in counts of anaerobic bacteria of the oral microflora in-flight compared to pre-flight samples. None of these changes were, however, deemed hazardous to astronauts’ health. In-flight increments of dental plaque, calculus, and gingival inflammation were moderate.	There was a sparsity of preflight and postflight clinical problems.	Culture-dependent assessment	Brown (1976) [90]
Sampling campaigns carried out for the Skylab program. Samples were obtained immediately before and after each Skylab mission.	Neck, ears, axillae, hands, navel, groin, toes, nose, throat, gargle, urine, feces.	n.a.	Decrease in the diversity of the microbial communities, although the overall microbial count went up following space flight. Inter-crew transfer of pathogens.	Culture-dependent assessment	Taylor et al. (1971) [91]
Mice were exposed to low LET γ radiation and high dietary iron, high LET ^38^Si particles, and spaceflight (for 13 days).	Colonic mucosa	Low LET radiation, IRON, and spaceflight induced distinct shifts in bacterial populations, but did not significantly elevate pathogenic genera.	n.a.	16S rRNA gene amplicon sequencing	Ritchie et al. (2015) [92]
Mice were exposed to high LET radiation.	Gut (fecal samples)	Substantial changes in the composition and functional potential of the gut microbiome, accompanied by changes in the abundance of multiple metabolites.	A distinct reorganization of the microbiota was observed at different doses as soon as 10 days post-radiation.	16S rRNA gene amplicon sequencing	Casero et al. (2017) [93]
520-day ground-based analog mission within an analogue Mars-surface habitat involving 6 crewmembers (MARS500 study). Analyses started before spaceflight and continued for 6 months after landing.	Gut (fecal samples)	Confinement determined a significant degree of temporal variability in the intestinal macrobiota. Individual specificity of the microbiota compositional layout was not compromised, however some key microbial components showed conserved temporal dynamics, with potential implications for the maintenance of a health-promoting, mutualistic microbiota configuration.	At the end of the mission, a return to the initial microbiota configuration was observed only in samples from 2 subjects, while new steady states were consolidated for the other crewmembers.	16S rRNA gene amplicon sequencing	Turroni et al. (2017) [94]
105-day analog mission at the Chinese Lunar Palace 1, involving 3 crewmembers.	Gut (fecal samples), habitat environmental (air filters)	Observed convergence in the microbiota composition of crew members reflected the common living environment and lifestyle. The bioregenerative life-support system (BLSS)—dietary structure determined an increased intestinal microbiome diversity and richness.	Intestinal microbiome diversity reverted to pre-experiment levels.	16S rRNA gene amplicon sequencing	Hao et al. (2018) [95]
Mice were exposed to hypergravity (3G) for 21 days.	Caecaland colonic samples	Hypergravity influenced intestinal microbiota composition, but without alteration in mucosal integrity.	n.a.	16S rRNA gene amplicon sequencing	Alauzet et al. (2019) [96]
Comparative study of an astronaut who joined a 1-year mission on the ISS, and his identical twin who remained on Earth. (Twins Study.)	Gut (fecal samples). Various other health parameters were also measured	Gut microbiota composition and function changed during spaceflight, but microbiome diversity remained unchanged.	Changes dissipated within a few weeks from landing.	Shotgun metagenome sequencing of genomic DNA	Garret-Bakelman et al. (2019) [97]
Mice were flown on the ISS for 37 days.	Gut (fecal samples)	Gut macrobiome structure was altered during spaceflight. Richness of the microbial community was unchanged.	n.a.	16S rRNA gene amplicon sequencing	Jiang et al. (2019) [89]
9 Crewmembers on a 6- to 12-month mission on the ISS. Sampling began 240 days before flight to establish a baseline of microbiome variability and content.	Gut (fecal samples), skin, nose, tongue	Spaceflight-dependent changes in the microbiome associated with the gastrointestinal tract, skin, nose, and tongue. Individual differences were observed in skin samples. The composition of the gut microbiota became more similar across astronauts in space, mostly due to a drop in the abundance of a few bacterial taxa.	Tongue: Many of the compositional changes reverted to preflight levels after the return to Earth. Nose, Gut and Skin: qualitative and quantitative changes in the microbial composition persisted for ~ 2 months in postflight samples.	16S rRNA gene amplicon sequencing	Voorhies et al. (2019) [98]
1 crewmember on a 135-day mission on the ISS. Samples were collected at 8 time-points pre-, during and post-flight.	Skin, nose, ear, saliva, habitat environmental (surfaces)	The microbiome of ISS surface environment resembled those of the astronaut’s nostril, ear, and in particular skin. Saliva microbiome diversity decreased during flight.	Saliva microbiome rebounded after returning to Earth.	Shotgun metagenome sequencing of genomic DNA	Avila-Herrera et al. (2020) [99]
Short-term space missions of 15 and 35 days involving 5 crewmembers. Sampling included the period before and after spaceflight.	Gut (fecal samples)	Short spaceflight markedly affected the composition and function of the human gut microbiota; however, the steady states of individual specificity could always be identified. These changes were accompanied by fluctuations in virulence and antibiotic resistance genes and in mobile genetic elements.	After four weeks’ recovery, the characteristics of samples was similar to the pre-flight samples.	DNA HiSeq sequencing	Liu et al. (2020) [81]
4 crewmembers on a 6-month mission on the ISS. Samples were collected at 8 time points pre-, during and post-flight.	Saliva and body swabs	Microbiome experienced a change in composition during spaceflight, but these changes were not universal for all four astronauts. Two antimicrobial resistance gene markers did show a significant change in abundance in the saliva samples of all four astronauts across their collection times.	Changes in microbial diversity were not permanent and returned to pre-flight levels after returning to Earth.	Shotgun metagenome sequencing of genomic DNA and microarrays.	Morrison et al. (2020) [100]
10 male crewmembers on a 2- to 9-month mission on the ISS. Samples were collected pre- during and post-flight.	Saliva	No population level differences were detected as a result of spaceflight. Half of the participants involved in the study, on their first flight, had distinct microbial communities pre-flight, in-flight, and post-flight. The other 5 subjects, who had previously flown to the ISS, did not have microbiome differences. A significant positive correlation between microbiome richness and EBV viral titers was observed.	Post-flight samples of the 5 subjects whose microbiome was not impacted by flight, were not similar to pre-flight samples even after 6 months from return.	16S rRNA gene amplicon sequencing, qPCR	Urbaniak et al. (2020) [101]
Reanalysis of the MARS500 project data from early (days 7–45) and late (days 420–520) fecal samples.	Gut (fecal samples), habitat environment (surfaces)	The reanalysis confirmed a significant alteration in the relative abundance of the microbiome throughout the period of the study. A certain level of species overlapping could be observed between the crewmembers and their habitat.	n.a.	Improved 16S rRNA gene amplicon bioinformatic technology	Brereton et al. (2021) [102]
4 crewmembers involved in a 180-day ground-based confined experiment in the Controlled Ecological Life Support System (CELSS).	Oropharyngeal and nasal swabs	The structure of the oropharyngeal and nasal microbiota changed during confinement. Despite individual differences, inter-individual transfer among occupants was observed.	An outbreak of *Rossella* happened 1–2 months after confinement, then it returned to normal.	16S rRNA gene amplicon sequencing	Chen et al. (2021) [103]

## Data Availability

Not applicable.
